# Supplementary material for: Microbial Ecological Signatures Predict Pathogen Emergence and Multidrug Resistance in Cystic Fibrosis Airways up to a Year in Advance
Source: medRxiv. 2026 Jan 2:2025.12.28.25342520. Preprint. [Version 1] doi: 10.64898/2025.12.28.25342520 (PMC12772665; doi:10.64898/2025.12.28.25342520)
Supplement: Supplement 1 [file media-1.pdf]

Microbial Ecological Signatures Predict Pathogen Emergence and Multidrug Resistance in Cystic Fibrosis Airways up to a Year in Advance

Goddard, Thomas R.[1,2,5,*], Carlson-Jones, Jessica AP.[3,*], Morton, Judith[4,5], Ooi, Chee Y.[6,7], Tai, Andrew[8,5], Warner, Morgyn S.[9], Wong, John[8], Evans, Ieuan ES.[10,11], Hopkins, Emily[4], Iredell, Jonathan R.[12,13], Jersmann, Hubertus PA.[8,14], Whiteson, Katrine L.[15], Bouras, George[16,3,5], Doane, Michael P.[3], Falk, Nicholas W.[3], Green, Renee[3], Grigson, Susanna R.[3], Mallawaarachchi, Vijini[3], Martin, Belinda[3], Roach, Michael J.[2], Ryan, Feargal J.[2], Tarasenko, Anita[3], Papudeshi, Bhavya[3], Drigo, Barbara[17], Giles, Sarah K.[3], Harker, Clarice M.[2], Hesse, Ryan D.[3], Hodgson, Riley J.[18], Hussnain, Art[19], Hutton, Abbey[3], Inglis, Laura K.[3], Keneally, Christopher[20], Kerr, Emma N.[3], Liddicoat, Craig[18], Peddle, Shawn D.[18], Watson, Carl D.[18], Yang, Qi[21], Decewicz, Przemysław[22], Speck, Peter G.[18], Mitchell, James G.[3], Dinsdale, Elizabeth A.[3], Edwards, Robert A.[3,†]

Supplemental Online Material

Supplemental Online Material 1
Supplemental Results Tables 2
Comparison of Sequencing Technologies 10
Supplemental Methods 11
Gradient Boosted Random Forest 11

Supplemental Results Tables

Table S1. Number of entries in the functional (subsystems) data at different levels and number of metagenome assembled genomes. We used the subsystems level for most analyses.

Hierarchical level	Number of entries
**Classes**	11
**Level 1**	29
**Level 2**	139
**Subsystems**	769
**All functions**	6,229
**Metagenome Assembled Genomes (MAGs)**	3,214

Table S2. Number of entries at different taxonomic levels for either just the Bacteria or all taxa (including Archaea, Bacteria, Eukarya, and Viruses). We used the Family level taxonomy for most analyses.

Taxonomy	Bacteria	All taxa
**Kingdom**	1	4
**Phylum**	164	252
**Class**	140	348
**Order**	302	837
**Family**	743	1,857
**Genus**	3,581	5,806
**Species**	26,499	31,371

Table S3. Features (taxonomy and subsystems) present in COPF 31, the most predictive cluster for *Pseudomonas* colonisation.

ABC-type iron transport system	Geothermobacteraceae	Potential DNA repair cluster
Acyclic terpene utilisation	Heme and heme d1 biosynthesis from siroheme	Pseudomonadaceae
Adenylylsulfate reductase	IbrA and IbrB: co-activators of prophage gene expression	Respiratory Systems - Shewanella (updated)
Aestuariirhabdaceae	L-2-amino-4-methoxy-trans-3-butenoic acid synthesis	Rhamnolipids in Pseudomonas

Beta-lactamases Ambler class D	Malonate decarboxylase	Salmochelin-mediated Iron Acquisition
Biogenesis of cbb3-type cytochrome c oxidases	Marinobacteraceae	Siderophore Pyoverdine
Cholera toxin	MexXY System of Pseudomonas aeruginosa	Stress proteins YciF, YciE
CMP-pseudaminic acid synthesis	Mycobacterial FadD proteins (fatty acid CoA- and AMP- ligases)	Sulfate assimilation related cluster
Coenzyme PQQ synthesis	Mycobacterial FadE proteins Acyl-CoA dehydrogenase	Sulfur transfer pathway CsdAEL
Colwelliaceae	Mycobacterial gene cluster associated with resistance against FAS-II antibiotics	Trans-envelope signaling system VreARI in Pseudomonas
Cytochrome c oxidase EC 1.9.3.1	Outer membrane porins in Pseudomonas and Acinetobacter	Two partner secretion pathway (TPS)
Extracellular matrix proteins (PEL) involved in glucose-rich biofilm formation in Pseudomonas	Periplasmic nitrate reductase EC 1.7.99.4	Type 4 conjugative transfer system, IncI1 type
Extracellular matrix proteins (PSL) involved in mannose-rich biofilm formation in Pseudomonas	Phenazine biosynthesis	Type III secretion system

Table S4. Features (taxonomy and subsystems) present in COPF 132, a predictive cluster for *Pseudomonas* colonisation.

Glycerolipid and Glycerophospholipid Metabolism in Bacteria	Cyanate hydrolysis	HipAB system implicated in growth arrest, persistence and drug tolerance
Hfl operon	One-carbon metabolism by tetrahydropterines	Toxin-Antitoxin system HigAB

Table S5. Features important for predicting MDR *Pseudomonas*

COPF	Features	Higher in
**29**	Carbonic anhydrase; Lipid orphans; P2-like phage, Universal stress protein family	Non-MDR
**68**	DNA processing cluster, Calvin-Benson cycle, Peptidase clustering with DAP, De Novo Pyrimidine Synthesis.	MDR
**82**	NADH ubiquinone oxidoreductase, Menaquinone biosynthesis from chorismate via 1,4-dihydroxy-6-naphthoate	Non-MDR
**18**	Fe-S cluster assembly, Streptococcal Hyaluronic Acid Capsule, Aspartate to Threonine Module, rRNA modification related cluster including tlyA, Translation termination factors, bacterial, RNA polymerase, bacterial, Sulfoquinovose biosynthesis cluster.	MDR

Table S6. Features important for predicting conversion to *Pseudomonas* culture positivity in the next 12 months.

COPF	Features	Higher in
**110**	Cell wall-associated cluster in Mycobacterium, 'Xanthine dehydrogenase subunits, Iron(III) dicitrate transport system Fec, Gemmatimonadaceae	
**24**	Phenylalanine and Tyrosine synthesis 1, DeNovo Purine Biosynthesis, Lysine DAP biosynthetic pathway, Diaminopimelate Synthesis, Lysine leader peptide	
**11**	Enoyl-[ACP] reductases disambiguation, NAD and NADP cofactor biosynthesis global, NAD and NADP cofactor biosynthesis bacterial, Fatty Acid Biosynthesis cluster, Resistance to Triclosan	

Table S7. Number of culture positive samples out of 127 samples in total

Culture Status	Number of positive samples
Achromobacter xylosoxidans	3
Acremonium species	1
Aspergillus flavus	3
Aspergillus fumigatus	22
Aspergillus nidulans	2
Aspergillus niger	1
Aspergillus terreus	1
Burkholderia cepacia	1
Candida albicans	40
Haemophilus influenzae	1
Inquilinus limosus	1
Lomentospora prolificans	1
Oral flora	31
Penicillium	3
Scedosporium apiospermum	1
Staphylococcus aureus	28
Stenotrophomonas maltophilia	9
**Pseudomonas**	
Pseudomonas aeruginosa	36
MDR *Pseudomonas aeruginosa*	14
mucoid	19
non-mucoid	13

Table S8. *Mycobacteria* observations in our cohort

pwCF Identifier	Days Since First Sample	Smear status	Cultured *Mycobacteria*
642660	256	negative	*M. abscessus; M. intracellulare*
670829	13	positive	*M. abscessus*
698564	443	negative	Unclassified rapid NTM (smear negative) - previously identified as *M. abscessus* (smear negative)
698917	80	negative	*M. abscessus*
717449	261	positive	*M. abscessus*
720054	7	negative	*M. intracellulare*
720054	63	negative	*M. intracellulare*
753522	261	positive	*M. abscessus*
763742	7	negative	unspecified NTM
770560	449	positive	*M. abscessus*
1162967	120	negative	*M. terrae* (intermediate grower)

Table S9. The *Aspergillus* culture status and sequence detection of pwCFID 676138 over almost 200 days.

		Metagenome sequence abundances[1]							***Culture Status***					***Aspergillus fumigatus culturing***	
**Days Since First Sample**	**Sum of antifungals**	***A. candidus***	***A. fischeri***	***A. fumigatus***	***A. mulundensis***	***A. sclerotialis***	***A. turcosus***	***A. udagawae***	***A. fumigatus***	***A. flavus***	***A. nidulans***	***A. niger***	***A. terreus***	**Previous 12 months**	**Next 12 months**
13	0	0.0	0.0	94.95	0.0	0.0	0.0	1.76	-	-	-	-	-	Yes	Yes
21	0	0.0	0.41	2.86	0.0	0.0	0.0	0.0	Yes	-	-	-	-	Yes	Yes
134	2	0.0	0.0	18.37	0.28	0.0	0.0	0.0	-	-	-	-	-	Yes	Yes
189	1	0.0	0.0	30.44	0.0	0.0	0.0	0.0	Yes	-	-	-	-	Yes	Yes
199	2	0.17	0.0	1.74	0.0	0.17	0.52	0.0	-	-	-	-	-	Yes	Yes

[1]Normalised abundance of each taxa identified from the short-read sequences.

Table S10. Allergic Bronchopulmonary Aspergillosis status for our cohort

ABPA status	Samples	Unique pwCF
**Not sensitised**	18	15
**Sensitised**	26	18
**ABPA**	7	4

Table S11. Accuracy of predicting *Pseudomonas* culturability in worldwide samples.

Project Title	Sample	Runs Analysed	Runs Correctly Predicted	True Positive Predictions (TP)	False Negative Preductions (FN)	True Negative Predictions (TN)	False Positive Predictions (FP)
Sputum Metagenome Of CF, COPD, Smokers And Healthy Subjects	PRJNA316588	17	17	2	0	15	0
Pseudomonas aeruginosa. Sinus, throat, and sputum airway microbiology in cystic fibrosis	PRJNA1081394	330	311	310	19	1	0
Depletion of human DNA from complex clinical samples for metagenomic sequencing	PRJNA516442	15	15	8	0	7	0
Shotgun metagenomic data from bronchoalveolar lavage fluid (BALF) samples of infants with CF	PRJNA1126024	2	2	1	0	1	0
Impact of ELX/TEZ/IVA therapy on the CF airway metagenome	PRJEB51171	9	8	3	1	5	0
Time series of patients with cystic fibrosis	PRJNA510441	14	5	5	9	0	0
Pseusomonas aeruginosa metagenomes	PRJEB14440	5	5	5	0	0	0
Microbiome analysis in the respiratory tract of individuals with cystic fibrosis and non-cystic fibrosis bronchiectasis	PRJNA1055940	61	45	9	16	36	0
Lung Microbiome Dynamics in Cystic Fibrosis	PRJEB32062	25	24	16	1	8	0
Study of fungal clinical isolates from different anatomical sites and metagenomics of the lung microbiome of patients with cystic fibrosis	PRJNA644285	12	11	3	1	8	0
The metagenome of children with cystic fibrosis	PRJNA931830	260	259	11	0	248	1

Total: Confident samples	750	702	373	47	329	1

Table S12. Accuracy of predicting *Pseudomonas* culturability in worldwide samples with uncertain provenance.

Project Title	Sample	Runs Analysed	Runs Correctly Predicted (TP)	Comments
Longitudinal changes on the oropharyngeal metagenome among children with cystic fibrosis after ETI	PRJNA1101448	323	-	It is not clear how many have Pseudomonas
Cystic fibrosis pathogens	PRJEB20836	8	8	
Viral and Microbial Cystic Fibrosis Lung Metagenome	PRJNA71831	21	4	It is not clear which samples, if any, have Pseudomonas
Sputum Metagenomics	PRJEB54014	75	21	There are no culture reports described in the paper
Cystic fibrosis airway respiratory microbiome	PRJNA615628	71	2	They culture Pseudomonas from 8 samples but do not specify which samples.
CF airway microbiome	PRJNA825831	114	12	In the paper they report 47% Pseudomonas positivity, but also describe 20/22 pwCF with Pseudomonas
Longitudinal Cystic Fibrosis Airway Raw sequence reads	PRJNA846291	98	1	No comment in the paper on positivity
Cystic Fibrosis Lung Sputum Microbiome before and after antibiotic treatment	PRJNA839435	12	11	There is no connection between SRA IDs and the IDs in the paper
Time-resolved study on cystic fibrosis airways	PRJNA516870	67	27	Unpublished
Metagenomic data from sputa from cystic fibrosis patients	PRJNA1091195	22	12	Unpublished
Sputum samples from cystic fibrosis patients' metagenomes	PRJNA316056	12	6	Unpublished
**Total: Samples with uncertain provenance**		**824**	**104**	

Comparison of Sequencing Technologies

We compared the taxa identified using atavide with the different sequencing technologies (Fig. S1). The companion notebook is available as a Jupyter notebook at MGI-MinION-Promethion_Compared.ipynb. We only show the data from the ten samples that were sequenced using all three technologies (x-axis). The normalised taxa from the MGI read abundance is shown on the y-axis. We filtered the data so that only those taxa with at least 10 reads per million mapped reads are shown, which omits the long tail of organisms that have very few reads mapped and that maybe of spurious value.

Supplemental Methods

Gradient Boosted Random Forest

Gradient-boosted random forests were trained with early stopping to avoid overfitting, with training halted when no further improvement in validation loss was observed.

Table S13. Gradient boosted random forest metrics.

Prediction	# Negative samples	# Positive samples	Early Stopping	Model MSE†
***Pseudomonas* culture positivity**	91	36	349	0.19
**MDR Pseudomonas**	22	14	100	0.375
**Conversion to *Pseudomonas* culture positivity**	38	36	11	0.67
**Mucoid *Pseudomonas* status**	10	16	100	0.5

†Mean squared error

The mean squared error (MSE) for our gradient-boosted models varied widely and are reported in the table above. The MSE provides a measure of residual variance: values closer to zero indicate tighter predictions around the true outcome, whereas higher values suggest weaker fit. In our approach, these differences largely reflect the type of outcome being modelled and the degree of biological signal present in the latent clusters. Predictions of strongly constrained outcomes, such as specific antibiotic exposures, yielded very low MSEs (0.01–0.23), whereas more diffuse or heterogeneous phenotypes (e.g. complex clinical states) naturally produced higher errors.

Higher MSEs indicate that the phenotype is only partially explained by the microbiome features captured here. Given the modest cohort size, the high dimensionality of the input space, and the intrinsic stochasticity of microbial communities, this variability is expected. However, the signal is consistent across repeated runs, feature selection using the autoencoder latent dimensions is stable, and the ROC curves show discrimination above chance. Instead, the MSE reflects the biological heterogeneity of the outcomes being modelling. Strongly predictive traits give tight errors; diffuse traits produce looser fits.
