# Supplementary material for: Microbial Ecological Signatures Predict Pathogen Emergence and Multidrug Resistance in Cystic Fibrosis Airways up to a Year in Advance
Source: medRxiv. 2026 Jan 2:2025.12.28.25342520. Preprint. [Version 1] doi: 10.64898/2025.12.28.25342520 (PMC12772665; doi:10.64898/2025.12.28.25342520)
Supplement: 2 [file NIHPP2025.12.28.25342520v1-supplement-2.pdf]

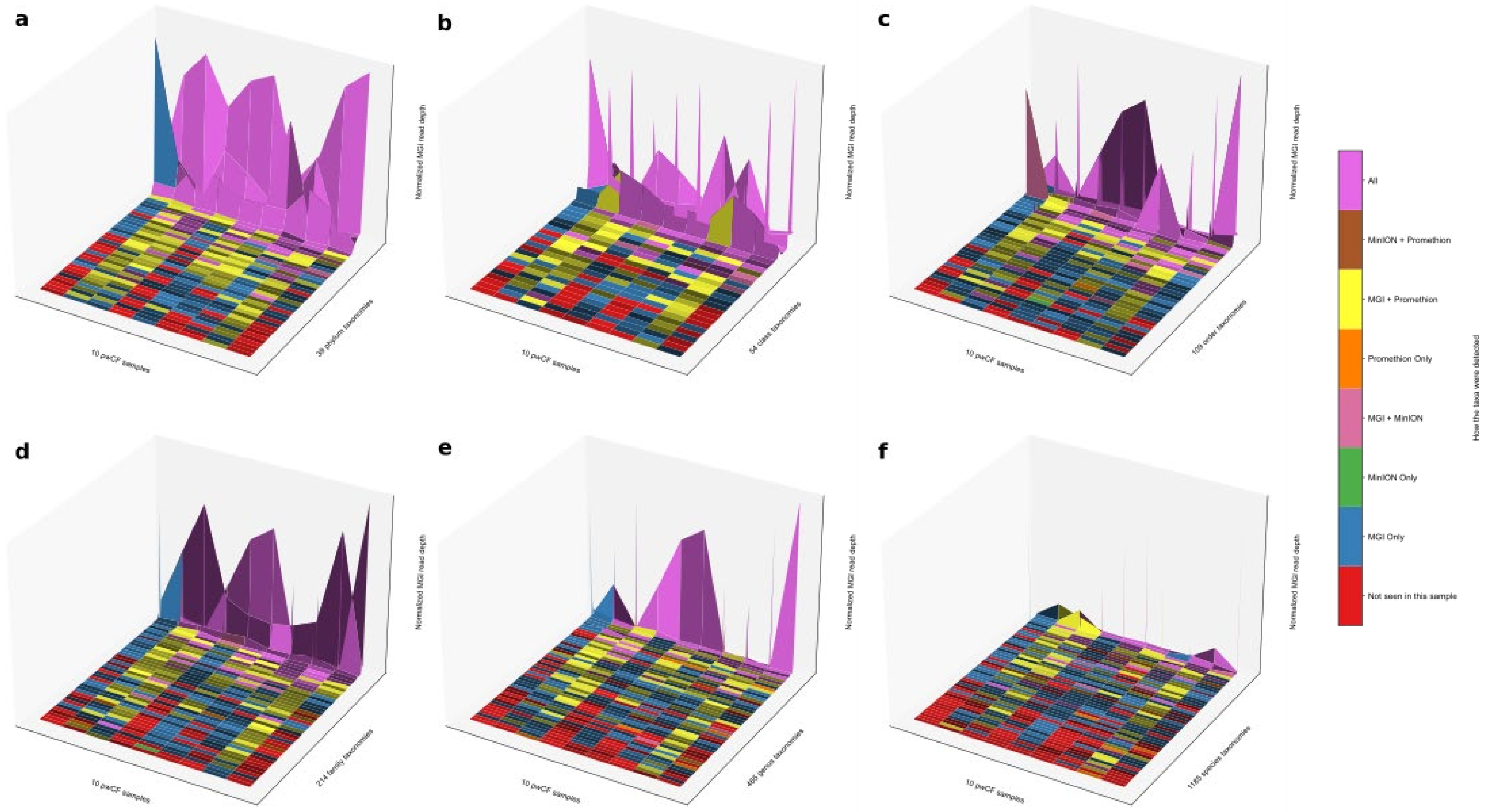

Supplementary Fig. 1. Comparison of species identified using MGI short read, Oxford Nanopore Minion, and Oxford Nanopore PromethION sequencing. The *n* = 10 samples that were sequenced using all three technologies are shown on the x-axis, the different taxa (unlabelled) are shown on the y-axis, and the normalised MGI read abundance is shown on the z-axis. Only those taxa with at least 10 reads per million mapped reads are shown. Colours indicate whether the taxa was identified with any of the sequencing technologies. Each panel reflects a different taxonomic rank: a. 39 phyla, RPKM ranges up to 811,485; b. 54 classes, RPKM ranges up to 781,208; c. 109 orders, RPKM ranges up to 694,463; d. 214 families, RPKM ranges up to 636,830; e. 465 genera, RPKM ranges up to 597,643; f. 1,185 species, RPKM ranges up to 388,863.

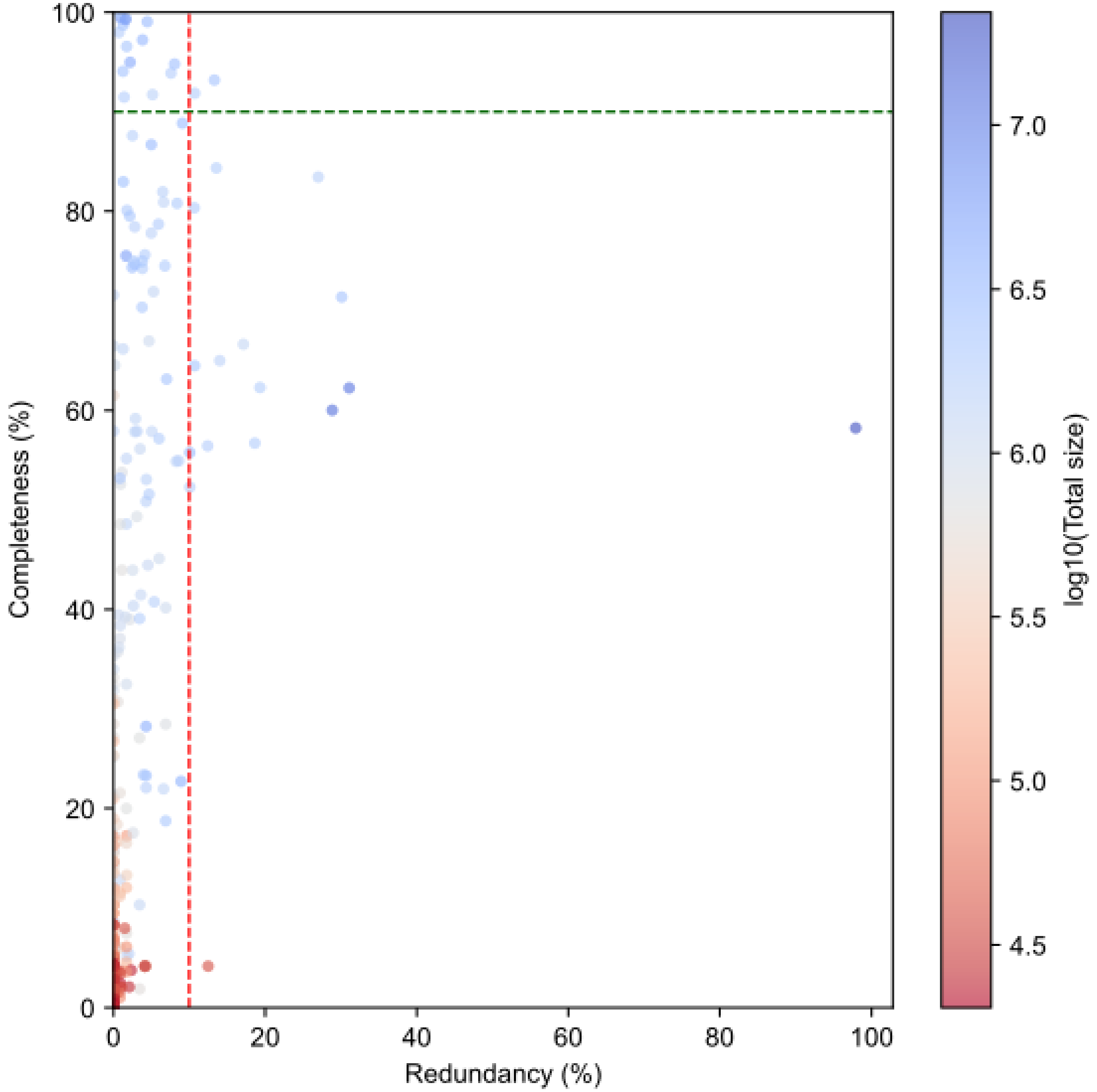

Supplementary Fig. 2. Relationship between completeness, redundancy, and total assembled genome size across all metagenome-assembled genomes (MAGs). Each point represents a MAG, positioned by its redundancy (x-axis) and completeness (y-axis), and coloured by the $\log_{10}$ of its total assembly size. Dashed lines mark thresholds commonly used to define high-quality metagenome-assembled genomes (≥90% completeness and ≤10% redundancy).

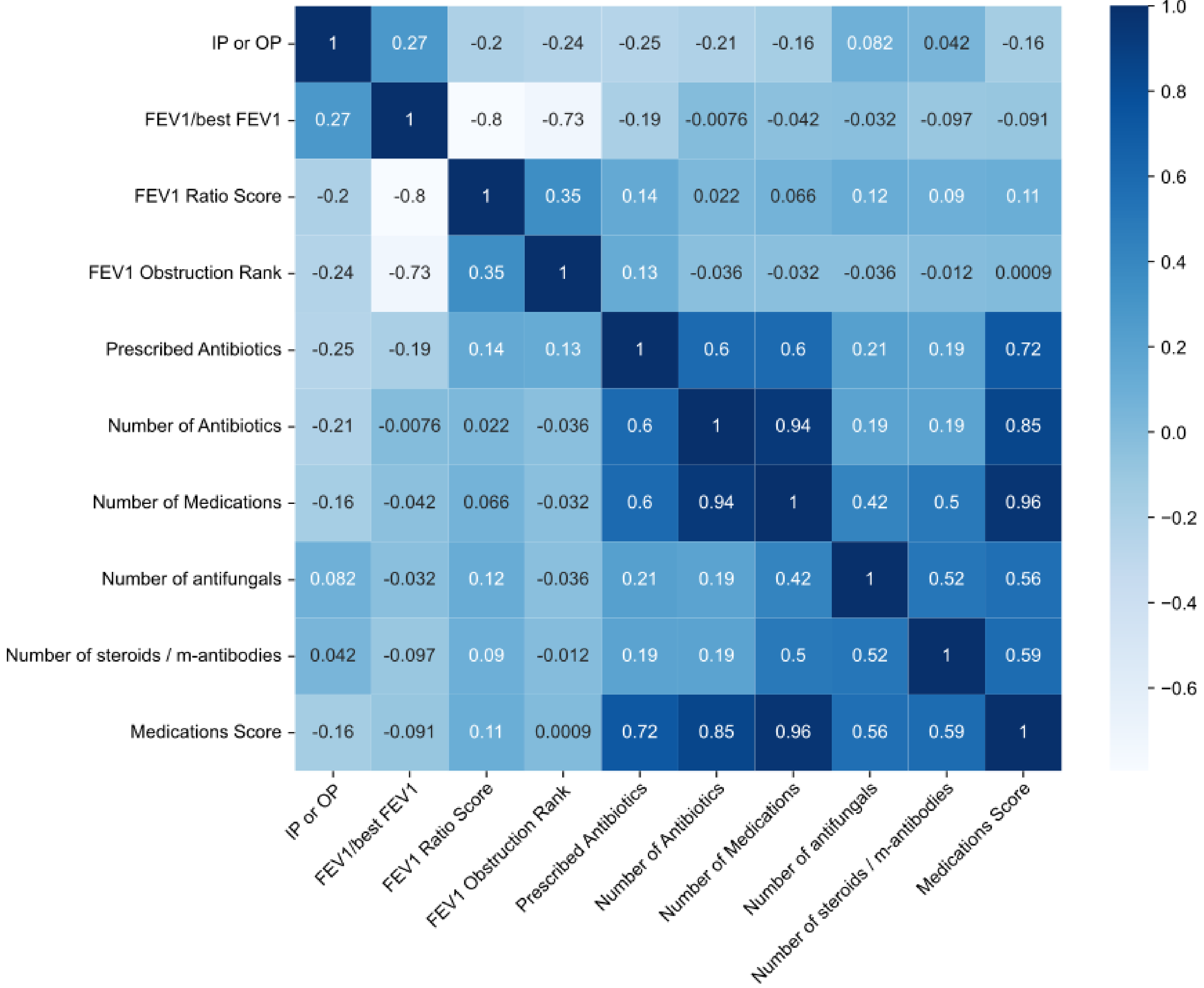

Supplementary Fig. 3 Pearson correlation of Medications Score and independent clinical measures. Pearson correlation matrix of the Medication Score and independent clinical measures. Each box is shaded by the strength and direction of the linear correlation (Pearson r), with the corresponding r-value shown inset (*n* = 125).

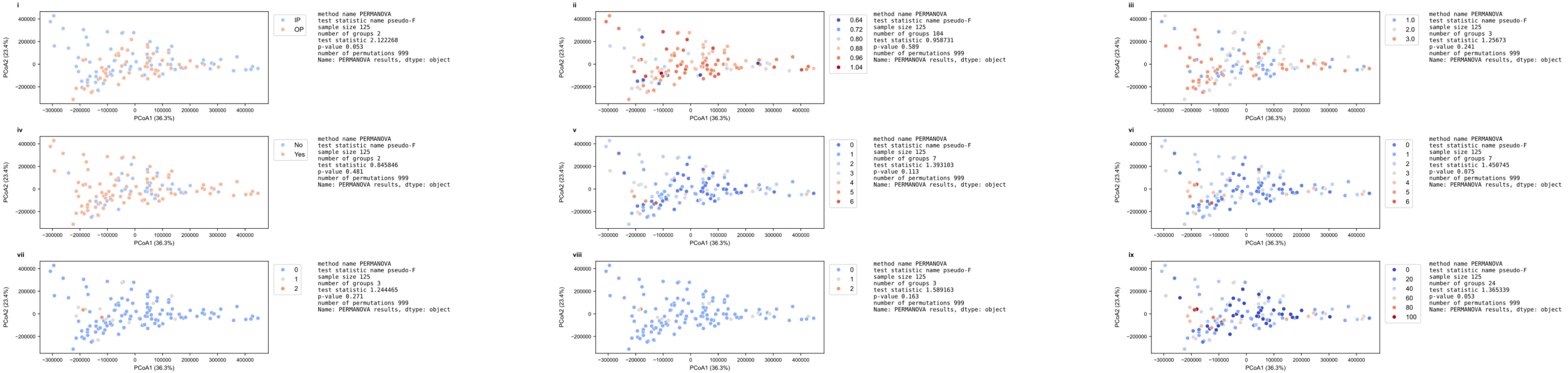

Supplementary Fig. 4 PCoA of the CPAF coloured by health measure. Each plot is the same scatterplot; however, we have coloured each point by the respective health score. We used PERMANOVA to calculate the clustering's statistical significance (pseudo-*F*) shown to the right of the figure. i) in-patient vs outpatient (p=0.07); ii) FEV_1/best FEV_1 (p=0.96); iii) FEV_1 ratio score (p=0.22); iv) Whether they were prescribed antibiotics (p=0.53); v) How many antibiotics they were prescribed (p=0.12); vi) How many medications they were prescribed (p=0.08); vii) How many antifungals they were prescribed (p=0.26); vii) How many steroids and monoclonal antibodies they were prescribed (p=0.17); ix) Medications Score (p=0.03**) (*n*=125 for each panel)

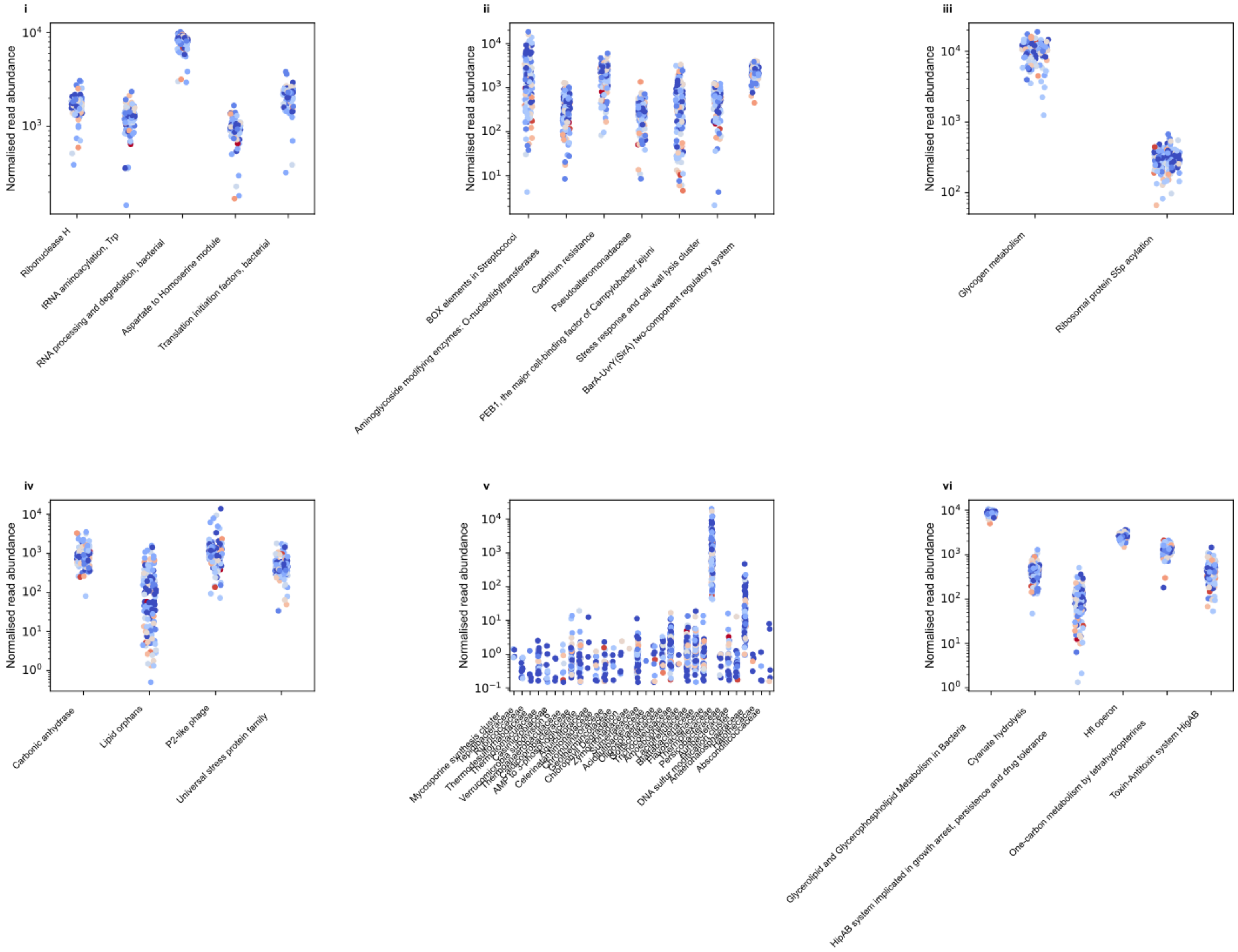

Supplementary Fig. 5. The normalised read abundance for each of the functions and phylogenies. The abundance is coloured by medication score from blue (least) to red (highest medication score). i) COPF 14; ii) COPF 6; iii) COPF 107; iv) COPF 29; v) COPF 138; vi) COPF 132 (*n* = 125 for each panel).

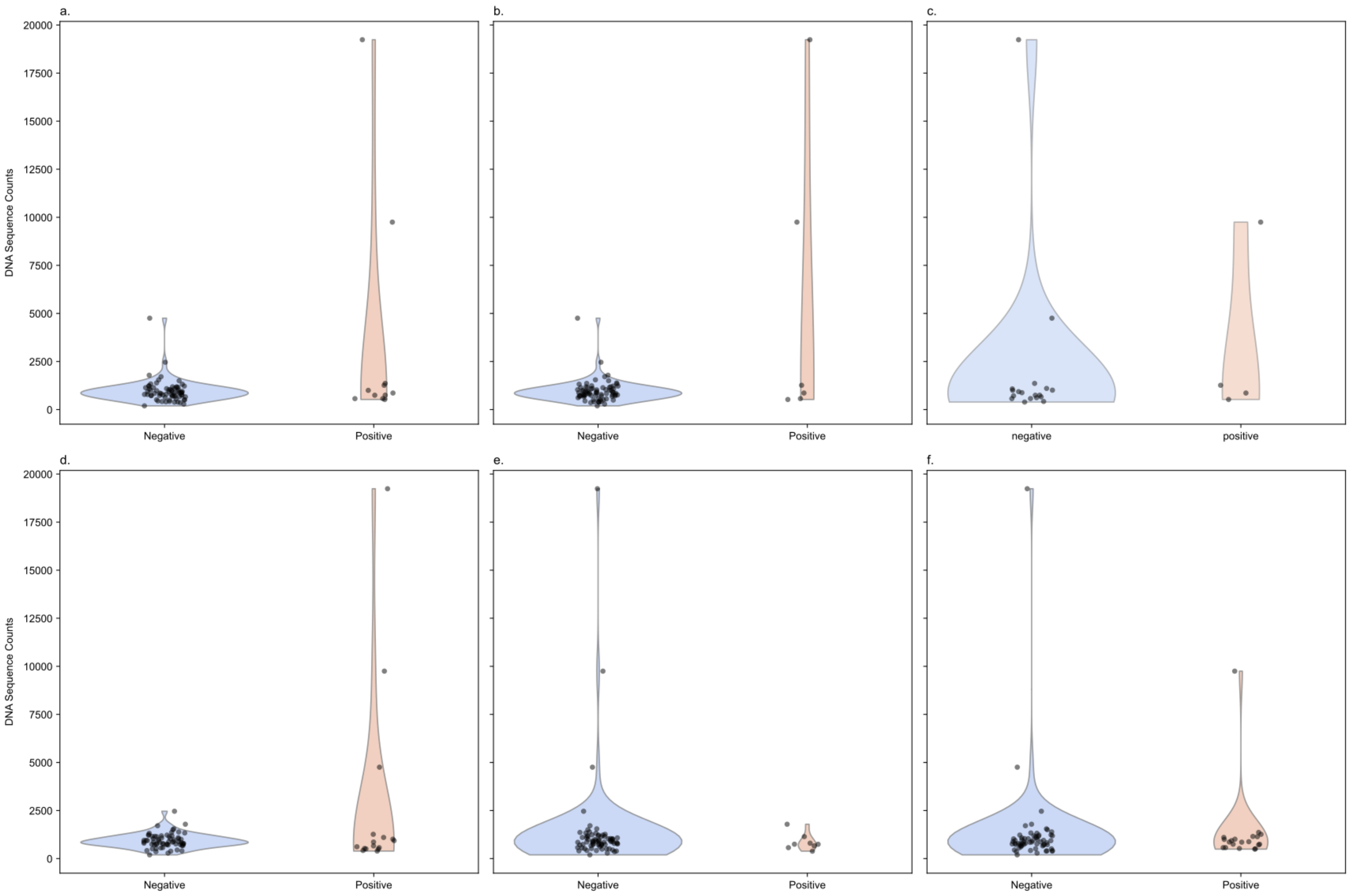

Supplementary Fig. 6. Culture of NTM compared to number of sequences detected. a. NTM culture status (n=82), **b**. *Mycobacteroides abscessus* culture status (n=82), **c.** NTM smear status (n=22), **d.** Rapid-growing NTM culture within the past year (n=82), **e.** Slow-growing NTM culture within the past year (n=82), **f.** Positive over the next 12 months (n=82)

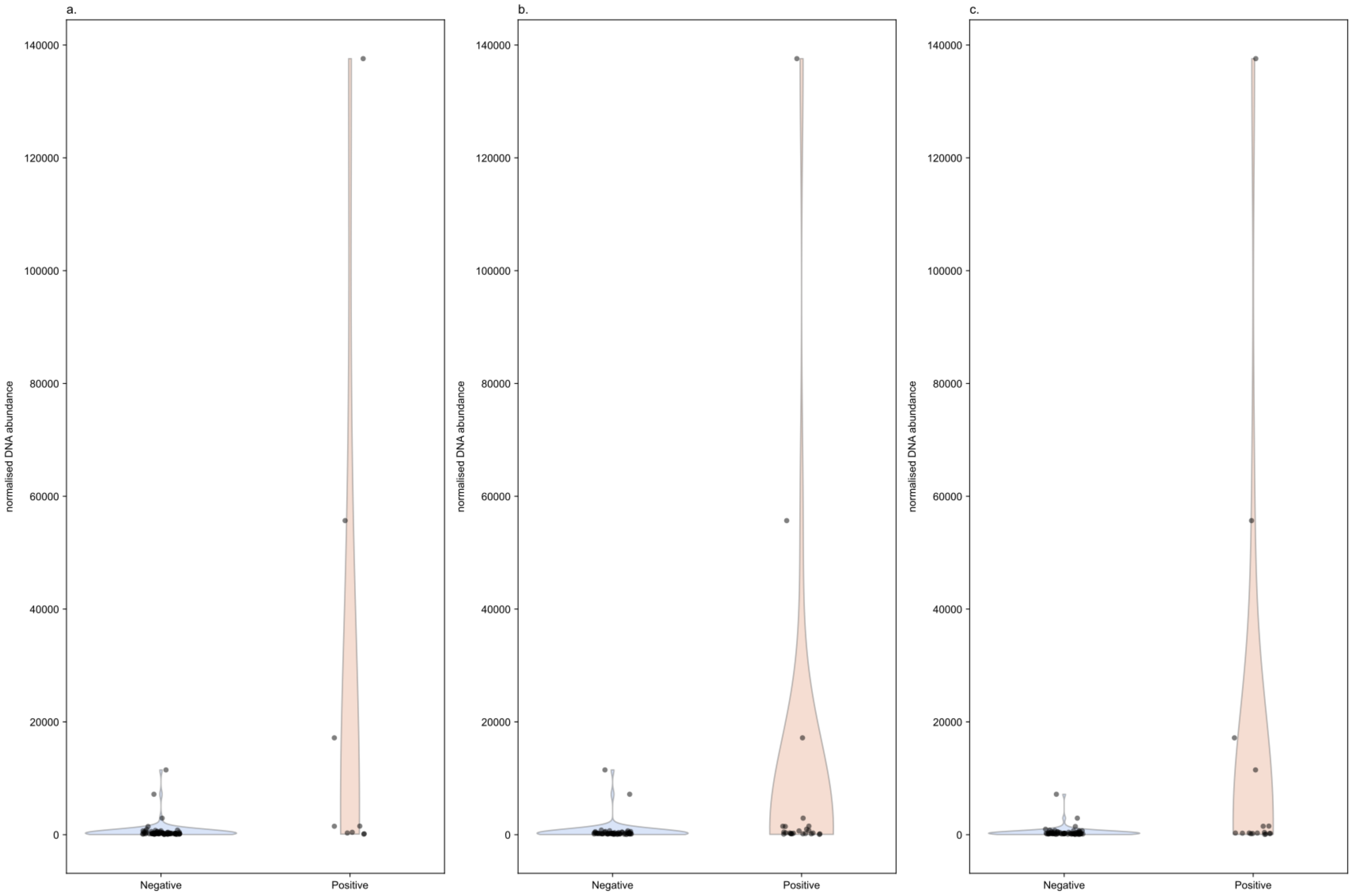

Supplementary Fig. 7. *Stenotrophomonas* culturing vs. sequence read abundance a. *Stenotrophomonas maltophilia* culture status, **b.** *Stenotrophomonas maltophilia* culture status in the previous 12 months, **c.** *Stenotrophomonas maltophilia* culture status in the next 12 months (n=81).

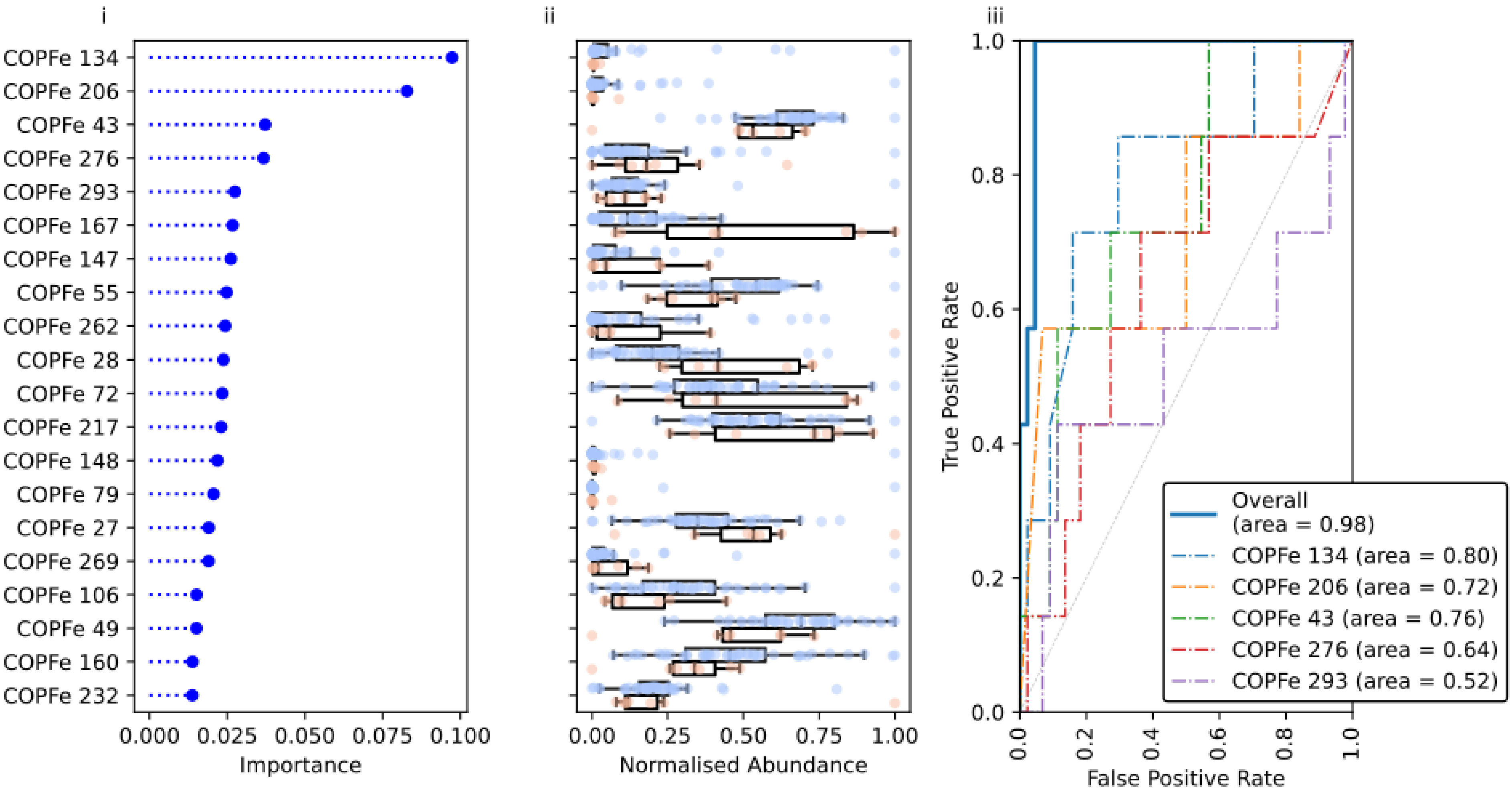

Supplementary Fig. 8. Detection of Allergic Bronchopulmonary Aspergillosis. The triptych contains (i) the gradient-boosted random forest feature importance plot for each eukaryotic COPF (called COPFe), (ii) the abundance of those COPFes in samples with (red) or without (blue) ABPA, and (iii) the ROC curve for the top 5 COPFes at predicting the phenotype and the overall ROC curve for all data. Area under the curves (AUC) are included in the ROC legend (n = 51).
